# Finding the Key Periods for Assimilating HJ-1A/B CCD Data and the WOFOST Model to Evaluate Heavy Metal Stress in Rice

**DOI:** 10.3390/s18041230

**Published:** 2018-04-17

**Authors:** Shuang Zhao, Xu Qian, Xiangnan Liu, Zhao Xu

**Affiliations:** 1 School of Information Engineering, China University of Geosciences, Haidian District, Beijing 100083, China; zhaoshuang316@163.com; 2 96669 Troops, Changping District, Beijing 102208, China; qqairforce@126.com; 3 State Grid Energy Research Institute, Changping District, Beijing 102209, China; xuzhao1@sgeri.sgcc.com.cn

**Keywords:** remote sensing, key period, heavy metal stress, WOFOST model, Harris algorithm, data assimilation

## Abstract

Accurately monitoring heavy metal stress in crops is vital for food security and agricultural production. The assimilation of remote sensing images into the World Food Studies (WOFOST) model provides an efficient way to solve this problem. In this study, we aimed at investigating the key periods of the assimilation framework for continuous monitoring of heavy metal stress in rice. The Harris algorithm was used for the leaf area index (LAI) curves to select the key period for an optimized assimilation. To obtain accurate LAI values, the measured dry weight of rice roots (WRT), which have been proven to be the most stress-sensitive indicator of heavy metal stress, were incorporated into the improved WOFOST model. Finally, the key periods, which contain four dominant time points, were used to select remote sensing images for the RS-WOFOST model for continuous monitoring of heavy metal stress. Compared with the key period which contains all the available remote sensing images, the results showed that the optimal key period can significantly improve the time efficiency of the assimilation framework by shortening the model operation time by more than 50%, while maintaining its accuracy. This result is highly significant when monitoring heavy metals in rice on a large-scale. Furthermore, it can also offer a reference for the timing of field measurements in monitoring heavy metal stress in rice.

## 1. Introduction

Along with the rapid development of industrialization and urbanization, soil contamination by heavy metals in China continues to worsen. Heavy metal contamination in the soil can accumulate in rice, which then threatens food security and damages human health [1,2,3,4,5]. Thus, it is extremely important to control heavy metal pollution in agricultural soil and monitor agricultural eco-environments.

Remote sensing plays an important role in monitoring heavy metal contamination. Numerous previous studies have investigated the relationship between spectral features (e.g., red edge position, spectral vegetation indices) and heavy metal concentrations or the physical characteristics of plants (e.g., leaf area index, yield, chlorophyll content) [6,7,8,9,10,11,12]. Many researchers have found that roots respond to heavy metal toxicity earlier and more strongly than the parts of the plant that are above ground [13,14,15,16,17]. Heavy metals can accumulate in roots because they are the entry point for water and nutriment into plants and can directly absorb heavy metals from contaminated soil. The dry weight of roots (WRT) is a representative indicator for monitoring heavy metal stress; however, the WRT is not able to be directly observed by remote sensing; Therefore, to continuously obtain dynamic WRT values, an optimized method that can assimilate remotely sensed data into improved crop growth models has been used [18,19,20,21,22,23,24,25,26,27]. The World Food Studies (WOFOST) model is a strong model that can describe the fundamental processes of crop growth, such as photosynthesis, respiration, transpiration and biomass partitioning [28]. For different areas, the relevant weather conditions, edatope, and crop parameters can be used to regionalize the model; thus, the model can simulate crop growth locally. The remotely sensed data assimilated in the WOFOST (RS-WOFOST) provide a reference method to dynamically monitor heavy metal contamination in paddy rice [18,19,20,21,22,23]. However, in previous studies, the key period used to assimilate remotely sensed data was not considered in the improved crop growth models [24,25,26]. It is very helpful to select key periods for the assimilation of remote sensing and the WOFOST model in continuous monitoring heavy metal stress in rice. Selecting key periods not only improves the accuracy and efficiency of the rice assimilation method applied at the regional scale, but also provides a time reference for the field measurements in monitoring stress. Heavy metal contamination has different effects during different plant growth periods [29,30]; hence, the time of the field measurement and monitoring is directly related to the effect of heavy metal stress and the accuracy of the assimilation model. Therefore, selecting the critical time is extremely important and necessary when monitoring heavy metal stress in rice.

In this study, the key period of RS-WOFOST assimilation was optimized based on the Harris algorithm to the original leaf area index (LAI) curves. The LAI is a botanical parameter that can be retrieved from remote sensing images [31] and it is also a key index for determining the appropriate period to monitor heavy metal stress in rice tissues. In order to improve the accuracy of LAI simulation, we embedded a specific stress factor (*f*) into the original WOFOST model. Moreover, the time-series WRT values were assimilated into the WOFOST model, and the stress factor was optimized in the process of assimilation. The modified WOFOST model was driven with the optimum (*f*) to realize the dynamic simulation of LAI. [24,26] The key period of the assimilation algorithm was analyzed using the Harris algorithm [32,33]. After screening, dominant time points were extracted and applied in the study area. Based on the key period, appropriate remote sensing images could be selected for the RS-WOFOST assimilation framework to realize the spatio-temporal continuous evaluation of heavy metal stress in rice tissues.

## 2. Study Area and Data

### 2.1. Study Area

The study area is located in Zhuzhou city, which is a primary grain production base for the Hunan Province, China. Historically, Zhuzhou is one of eight key national industrial cities. Therefore, with the development of industry, Zhuzhou has become an area with serious heavy metal pollution. The Xiangjiang River, which is the main irrigative river through the region, continually transports industrial pollutants to farmland [34,35,36]. Paddy rice is the main grain crop in Zhuzhou. Boyou9083 is the primary type of rice in this area.

**Figure 1:**
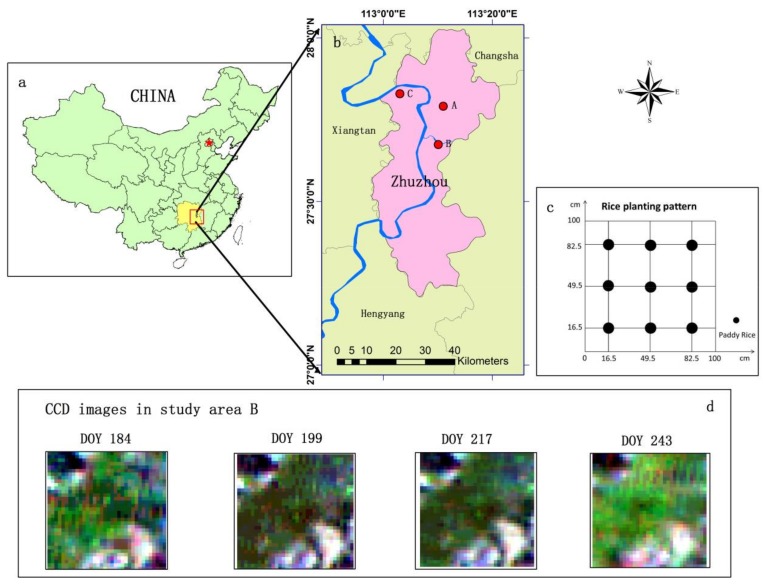
Location map for the study areas in the city of Zhuzhou, Hunan Province, China. DOY is the abbreviation for the day of year. (**a**) The location of Zhuzhou city in China; (**b**) the location of the study areas in Zhuzhou city; (**c**) diagram for the rice planting pattern; (**d**) the CCD images selected by the key periods in study area B.

Three subareas were selected (A, B, C) area A (113°12′30.38″ E, 27°47′31.01″ N) is located northeast of the Zhuzhou municipal districts and is far away from the Xiangjiang River; area B (113°10′18.65″ E, 27°40′28.35″ N) is adjacent to the Lujiang River, which is a tributary of the Xiangjiang River; area C (113°2′38.43″ E, 27°50′22.72″ N) is a rice paddy near the Xiangjiang River (Figure 1). The physicochemical characteristics of the soil were investigated for the three areas, and it was concluded that the heavy metal concentration of area A was lower than that of its background level, whereas the concentration was greater than the background levels in areas B and C (Table 1). According to the Environmental Quality Standard for Soils in China (GB15618-1995, EQSSC) and the pollution index, the Cd concentration in the Zhuzhou clearly exceeded these standards. With respect to local soil background values, these areas were classified as being at a “Safe” level, “Level I” or “Level II”. As environmental indicators, the mean annual temperature of the region is approximately 16–18 °C, and all of the areas belong to a subtropical monsoon climate zone with sufficient sunlight to grow paddy rice. The main soil type is orthicacrisol, which has a sufficient amount of organic matter (2–3%). The pH of the soil in the study area is between 5.0–8.5 [37]. To avoid other uncertain factors that might lead to growth stress, it was ensured that the paddy rice grew under adequate water with an irrigation channel system and timely fertilization.

### 2.2. Data Preparation

The data sets were collected from June to September 2014, including field data, remote sensing data, and meteorological data.

#### 2.2.1. Field Data

The field data contained soil and vegetation parameters for the entire rice growth period. In each subarea, 30 sampling points were equally distributed and surveyed on the four following dates: 4 July, (day of year (DOY) 185), 29 July (DOY 210), 28 August (DOY 240) and 17 September (DOY 260) [38]. The LAI of rice was measured by the botanical canopy analyzer (AccuPAR model LP-80). Rice and soil samples were collected, preserved in sample bags and dried at room temperature to achieve a constant weight, the collected soil samples were sited at the rooting zone according to the EQSSC, and the depth of the collected soil samples was 20 cm. Then the samples were delivered to the laboratory for testing and analysis. In particular, the dry weight of the rice roots (WRT) were calculated (Table 2), and the concentration of heavy metals in the soil and rice was measured using an atomic absorption spectrophotometer (AAS, Spectr AA 110/220, Varian, Palo Alto, CA, USA), and the nitrogen in the rice was measured by an elemental analyzer (Leco, San Jose, CA, USA) at the Chinese Academy of Agricultural Sciences [39] (Table 1).

#### 2.2.2. Remote Sensing Data

In this paper, the remote sensing images used were HJ-1A/B CCD data (Small Satellite Constellation for Environmental Disasters Monitoring and Prediction), which has a moderate spatial resolution (30 m), high time resolution (two days), large swath width (360 km or 720 km), and contains four spectral wavebands: 0.45–0.52 μm (blue waveband), 0.52–0.6 μm (green waveband), 0.63–0.69 μm (red waveband), and 0.76–0.9 μm (near-infrared waveband) [40]. Several CCD images that include the different growth periods of rice were selected to calculate the LAI. Radiometric calibration of the CCD images was performed using the calibration coefficient of each band provided by the China Centre for Resources Satellite Data. Then, the CCD images were geometrically corrected based on a rectified TM image, and atmospheric correction was conducted using the Second Simulation of the Satellite Signal in the Solar Spectrum (6S) model [41].

#### 2.2.3. Meteorological Data

In this study, meteorological data as input parameters were used for the WOFOST model. Meteorological data were acquired from the China Meteorological Data Sharing Service System and include the temperature, solar radiation and sunshine duration.

## 3. Method

**Figure 2:**
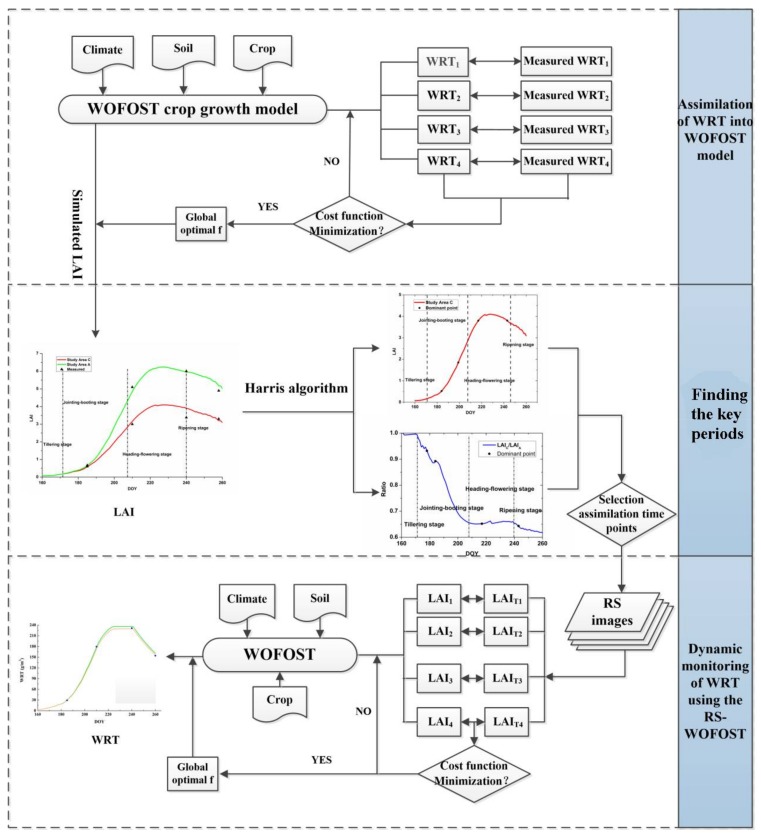
Flow chart of the WRT-WOFOST assimilation framework to find the key periods. (The WRT-WOFOST assimilation framework means assimilating the WOFOST model with WRT).

The key period can provide the basis for the timing of the selection of remote sensing images in the assimilation framework (Figure 2). Considering the various parameters of rice, LAI is a retrieved botanic parameter used for remote sensing, which serves as the observation data in the assimilation process [18,19,23,31]. The measured WRT was chosen as a state variable and used to modify the WOFOST model to simulate the continuous LAI under heavy metal stress. Then, the Harris algorithm was used on the original LAI curve in study area C and on the ratio of the LAI curve in study area A and C to select the optimized assimilation time points. Two different key periods were used to analyze the results. Finally, according to the key periods, the RS-WOFOST assimilation framework was established in study area B for monitoring heavy metal contamination in rice.

### 3.1. Simulating the Dynamic LAIs under Heavy Metal Stress

Data assimilation is a method that combines a model with observation data [20,42,43]. In this paper, the WOFOST model under potential production levels (suitable water and nutrients), was assimilated with the observation data. The WOFOST model is a model based on eco-physiological processes, which simulates the growth of crop by simulating the processes, such as CO_2_ assimilation, respiration, dry matter formation, growth of leaf area, transpiration, and phenological development [25,28]. The simulated LAI of the WOFOST model was computed by:

(1) $$\mathrm{LAI}\left(\mathrm{Iday}\right)=\;\overset{i=N-P}{\underset{i=1}{\sum }}\mathrm{LV}\left(i\right)*\mathrm{SLA}\left(i\right)$$

where SLA is the specific leaf area, SLA(i) is the specific leaf area whose leaf age is i. LV is the new leaf weight, LV(i) is the new leaf weight of the *i*th day, Iday is the *i*th day. N−P is the maximum leaf age of remaining leaves, more details refer the [28].

**Figure 3:**
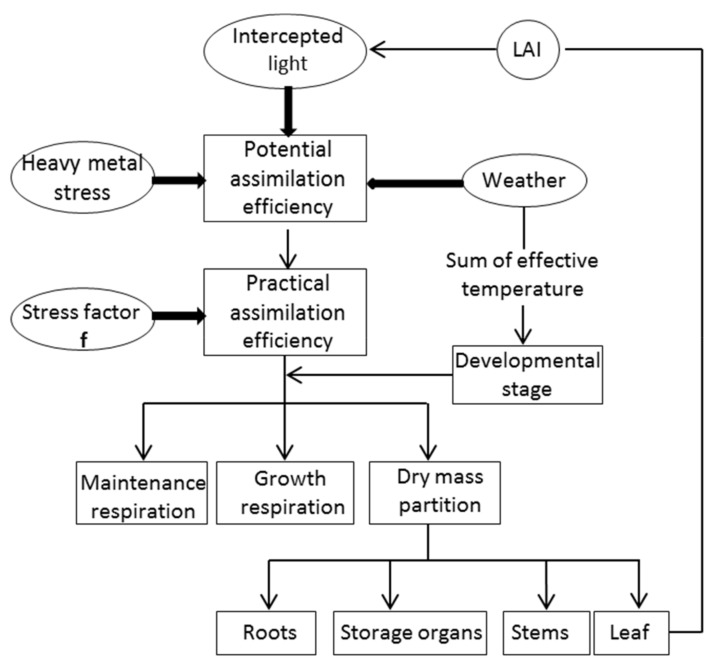
Simplified structure of the improved WOFOST model with stress factor *f*.

The WOFOST model was modified to determine the LAI under heavy metal stress [26]. To simulate the LAI more accurately, the WRT was chosen as an assimilation variable, i.e., as an output parameter of the WOFOST model, the WRT was optimized and revised by determining the optimal stress factor (*f*). Also the optimal stress factor (*f*) was obtained by minimizing the discrepancy between the simulated and measured WRT observations. Then, the stress factor *f* was embedded into the WOFOST model to simulate the dynamic LAI under heavy metal stress (Figure 3). The dynamic LAI values in area A and area C were calculated to analyze the key period.

To quantify the performance of the assimilation model, various important evaluation parameters were calculated, such as the correlation coefficient (R^2^) and the mean absolute error (MAE). The MAE indicated the absolute estimation errors. Fewer prediction errors were obtained with lower MAE values [44,45]. The two parameters were computed by:

(2) $$R^{2}=\frac{{\left[\sum_{i=1}^{n}\left(Y^{\prime }-\bar{Y}{}^{\prime }\right)\sum_{i=1}^{n}\left(Y-\bar{Y}\right)\right]}^{2}}{\sum_{i=1}^{n}{\left(Y^{\prime }-\bar{Y}{}^{\prime }\right)}^{2}\sum_{i=1}^{n}{\left(Y-\bar{Y}\right)}^{2}}$$

(3) $$\mathrm{MAE}=\frac{\sum_{i=1}^{n}\left|Y\prime_{i}-Y_{i}\right|}{n}$$

where $Y^{\prime }$ and Y are the predicted value and measured value, respectively, $\bar{Y}{}^{\prime }$ and Y are the average predicted value and the average measured value, respectively, and n is the number of samples.

### 3.2. Investigating the Key Periods Using the Harris Algorithm

The second moment matrix is the theoretical basis of the Harris detector. The second moment matrix, which is also called the auto-correlation matrix, is a measure to describe various combinations of adjacent pixels values in an image. Using this description method with the auto-correlation matrix, we can analyze the spatial correlation properties of pixels, describe the texture of an image, obtain distribution characteristics of pixel values and determine the statistical characteristics of changes in pixel values. The second moment matrix is defined by:

(4) $$M=\;\underset{x,y}{\sum }G\left(\tilde{\sigma }\right)\left[\begin{array}{cc} I_{x}^{2} & I_{x}I_{y}\\ I_{x}I_{y} & I_{y}^{2} \end{array}\right]$$

(5) $$G\left(\tilde{\sigma }\right)=e^{-\frac{x^{2}+y^{2}}{2{\tilde{\sigma }}^{2}}}$$

where $I_{a}$ is the derivative computed in the a direction, $G\left(\tilde{\sigma }\right)$ is the function of the Gaussian smoothing filter, and σ is the template size. The matrix describes the gradient distribution in the local neighborhood of a point. Gaussian kernels are used to compute the local derivatives. Then, a Gaussian window of size σ is used to smooth the derivatives, which are averaged in the neighborhood of the point. The Gaussian filter is programmed with template operations of various sizes (3 × 3, 5 × 5, 7 × 7, etc.); here, 3 × 3 templates will be used as an example. The average value of eight neighborhood pixels was used to replace the original value of the middle pixel to achieve the effect of smoothing. Two eigenvalues of the symmetric matrix M can be defined as $\lambda_{1}$ and $\lambda_{2}$ [33]. Therefore, the eigenvalues can represent two principal signal changes in the neighborhood of a point. The eigenvalues can help the extraction of points, where exist significant variation of curvatures, such as corner points and junction points. The Harris detector, which is one of the most popular interest point detectors, is also based on this principle [32,46,47]. Thus, the Harris measure combines the trace, where the determinant of the second moment matrix can be expressed as:

(6) $$R=\det M-k{\left(traceM\right)}^{2}$$

where $\det M=\lambda_{1}\lambda_{2}$, $\;traceM=\;\lambda_{1}+\lambda_{2}$,  k is an empirical constant, and the range of k is 0.04–0.06. Local maxima of R determines the location of interest point.

**Figure 4:**
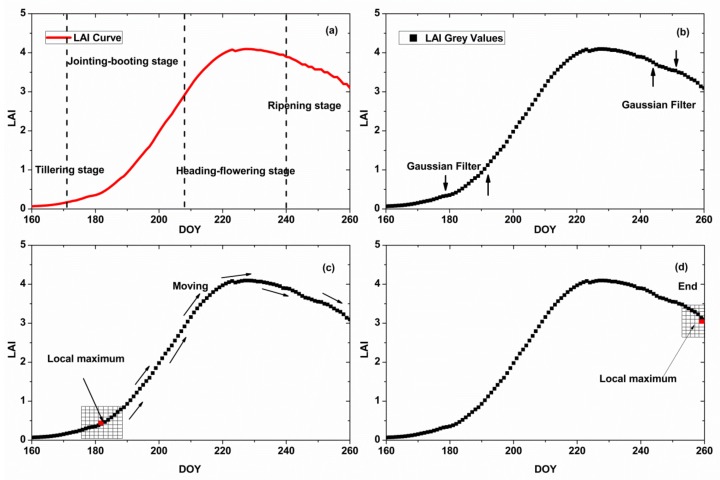
Harris algorithm process for the LAI curve. (**a**) is the original LAI curve; (**b**) is the smoothed LAI curve, the filter is Gaussian filter; (**c**,**d**) are the process of the Harris point detection.

**Figure 5:**
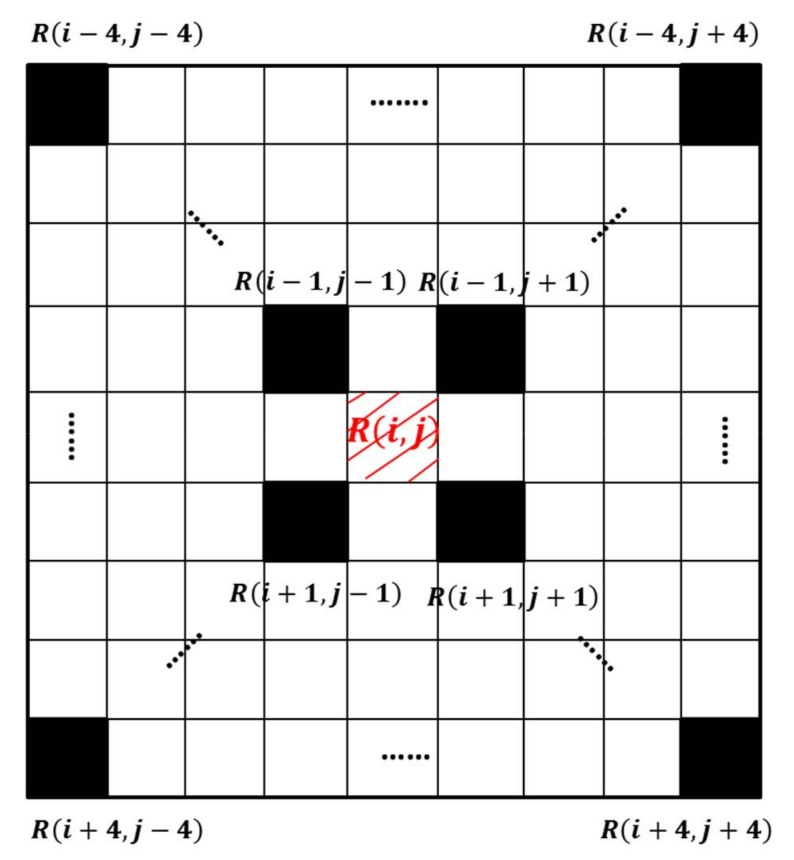
A 9 × 9 Gaussian window used to search for the dominant corner point.

Hence the Harris method is suitable for image detection, the LAI curve is converted to a grayscale image. The unit interval for the *X* axis is day, and the unit interval of *Y* axis is the minimum value of the LAI day increment (0.001). Then, Gaussian smoothing was conducted to remove noise points, as shown in Figure 4a,b. A Gaussian window of size 9 × 9 was used to detect the dominant point (Figure 5). The window was moved within the grayscale image to determine if the point is the dominant point or not, as shown in Figure 4c,d. The red shadow area is the center pixel of the window, which matches with the point in the grey curve. Using Formula (3)–(6), the calculated value of the center pixel is $R_{\left(i,j\right)}$; therefore, the values of the 9 × 9 window pixels were $R_{\left(i-4,j-4\right)}$ to $R_{\left(i+4,j+4\right)}$ from the top-left corner to the bottom-right corner, respectively.

The dominant corner point is the location of local maximum, i.e., if the center of window $R_{\left(i,j\right)}$ is greater than the other pixels of the window, the center point is the dominant corner point. Figure 5 shows the Harris algorithm process for the LAI curve, which searches for the dominant corner point. When meeting local maxima, the Gaussian window stops and records the location and then moves on. During the whole process, the dominant corner points were selected.

### 3.3. The Application of the Key Periods for the RS-WOFOST Model

The WRT, the plant organ most sensitive to heavy metal stress, is widely used in the monitoring of heavy metal contamination of rice [24,26]. To verify the appropriate monitor key period, the assimilation method was introduced to dynamically simulate the WRT. The unit of the WRT simulated by the WOFOST model is kilograms per hectare ($\mathrm{kg}/{\mathrm{hm}}^{2}$), which is too coarse for a parcel of the rice field in the study area. Thus, in this study, the unit was converted to grams per square meter ($g/m^{2}$). As illustrated in Figure 1c, it is assumed that the rice plants in the study areas are uniformly distributed, where there are nine paddy rice plants in one square meter. Then, the average WRT was multiplied by nine with the unit of grams per square meter as the measurement.

LAI is an important physiological-ecological parameter for crops and it is also a popular index in agricultural remote sensing research. Under the influence of heavy metal stress, the LAI clearly changes [26]. Furthermore, if LAI is an output parameter of the WOFOST, it can be retrieved by remote sensing images Therefore, the LAI was chosen as an assimilation variable to integrate the WOFOST model with remote sensing images.

LAI used as an assimilation variable is calculated from the CCD images. Many previous studies have shown that the normalized difference vegetation index (NDVI) is the most popular method to retrieve LAI from remotely sensed data [48,49]. However, when the LAI values increase, the NDVI tends to become saturated [50,51]. Therefore, to enhance the accuracy of the calculated LAI values, a new index, which is called GBNDVI (Green-Blue NDVI) and is an improvement of the NDVI, was determined [52]. GBNDVI avoids the saturation problem and can calculate LAI values well. Thus, GBNDVI was applied to retrieve the LAI in the study areas, which is expressed by:

(7) $$\mathrm{GBNDVI}=\frac{\left[IR-\left(G+B\right)\right]}{\left[IR+\left(G+B\right)\right]}$$

(8) $$\mathrm{LAI}=0.3243e^{5.3944*\mathrm{GBNDVI}}$$

where IR, G, and B represent the reflectivity of the near-infrared band, green band, and blue band in the CCD images, respectively.

The assessment of the accuracy of the method in this section is the same as the method described in Section 3.1.

## 4. Results

### 4.1. Performance of the Improved WRT-WOFOST Model

The WRT-WOFOST model was created to monitor the growth situation of rice under heavy metal stress. Before assimilating, the parameters of the WOFOST model were regionalized using climate data, soil data and rice growth data in the local area. Then, the WRT was measured during the different crop periods and was assimilated into the improved model. In this study, the PSO algorithm, which describes the process of assimilation, was used to optimize and iterate the WRT between the measured and simulated values [53]. The WRT assimilation cost function is as follows:

(9) $$P=\;\frac{1}{n}\sqrt{\overset{n}{\underset{i=1}{\sum }}{\left(WRT_{Mi}-WRT_{Si}\right)}^{2}}$$

where n is the number of WRT measured values, here, n is 3, $WRT_{Mi}$ and $WRT_{Si}$ are the measured data and simulated data of the *WRT* at the *i*th time, respectively.

In the 1 km ×1 km study area, there are other land types that are not used for rice planting. Hence, land classification was conducted before the assimilation process. After classification, the non-rice areas were excluded. Then, the WRT-WOFOST framework was applied to every pixel to obtain the stress factor (f). The stress factor (f) in the rice paddy of the two study areas describes the pollution level of each area (Table 3).

**Figure 6:**
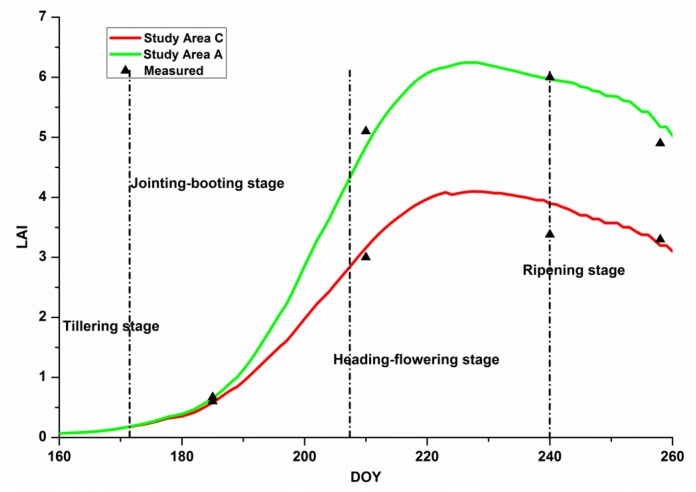
Dynamic simulation of the leaf area indexes (LAIs) by the optimized model for each study area.

The results indicate that the range of the stress factor agrees with the pollution level of each study area shown in Table 1. The heavy metal concentration of soil can influence the carbohydrate assimilation efficiency of rice [25,26]. Therefore, the more polluted a plant becomes, the smaller the f values. According to the above analysis, stress factor (f) has the potential to distinguish different heavy metal stress levels. Then, the stress factor is introduced into the WOFOST model to simulate the LAI during the entire rice growth period under heavy metal stress with a step-length of 1 day. The simulated LAIs by the optimized model for study area A and C were shown in the Figure 6.

To determine the key periods to monitor heavy metal stress, the accuracy of the WRT-WOFOST model should be verified. The R^2^ and MAE values were calculated by Formulas (1) and (2).

Table 4 shows that when the optimized WOFOST model is applied to the two study areas, both have high precision. In study area A, the R^2^ and MAE values are 0.988 and 0.214, respectively, and in study area B, the R^2^ and MAE values are 0.97 and 0.199, respectively. Hence, it can be concluded that the WRT-WOFOST model exhibits a good performance in monitoring rice growth under heavy metal stress and can be used to further analyze the key period of assimilation.

### 4.2. Determination of the Key Periods

The LAI curve under stress (area C) was used to extract the dominant points. Then, the ratio between the LAI in study area A and the LAI in study area C was calculated to extract the dominant point, the LAI in study area A was used as the background value.

**Figure 7:**
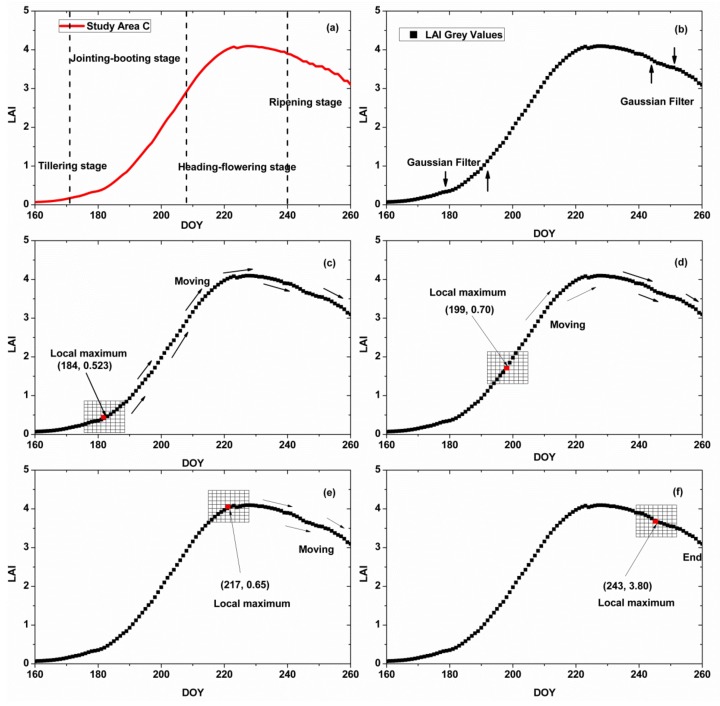
Process of the Harris algorithm of the LAI curve in study area C: (**a**) original LAI curve; (**b**) LAI curve with Gaussian filter; (**c**–**f**) process of searching for the dominant points in the LAI curve.

Figure 7 shows the process of the Harris algorithm in study area A, which searches for the dominant corner point. During all of the rice growth stages, four dominant corner points were selected.

**Figure 8:**
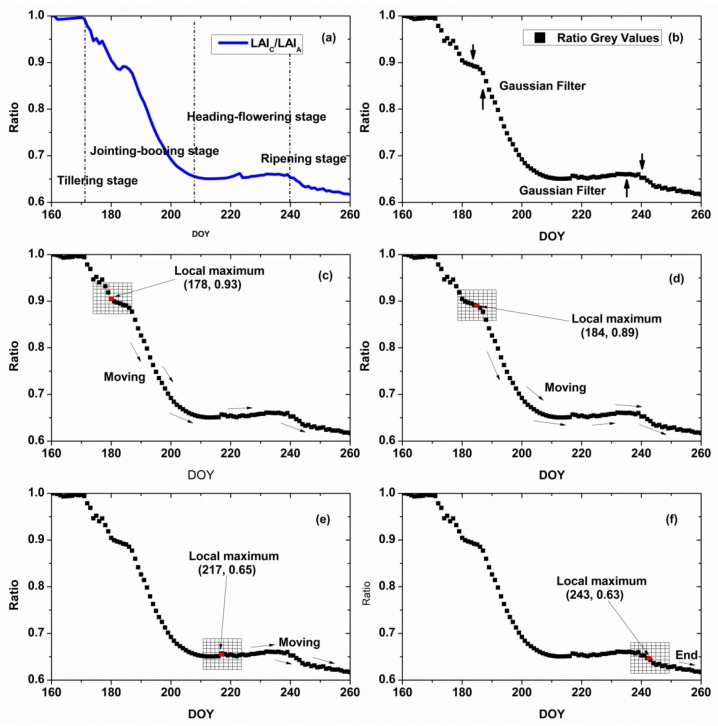
Process of the Harris algorithm in the ratio curve: (**a**) original ratio curve; (**b**) ratio curve with Gaussian filter; (**c**–**f**) process of searching for the dominant points in the ratio curve.

The ratio between the LAI values in study area A and C describes the difference between LAIs of two areas; therefore, in the selection of a dominant point, it is helpful to extract the corner point of the ratio. Similarly, with the LAI, the Harris algorithm was also used to search for this point. Figure 8 illustrates the process in detail, where four points were eventually selected. 

**Figure 9:**
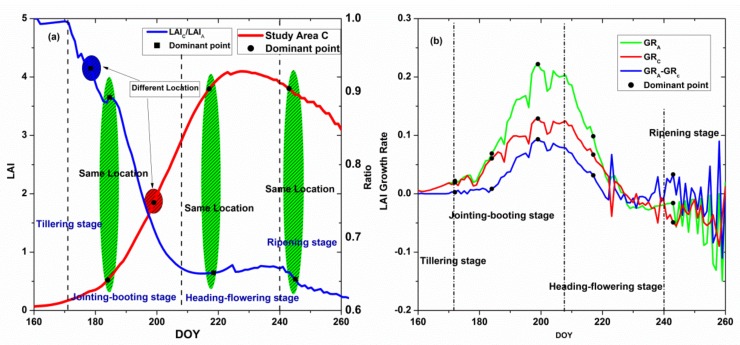
Dynamic assessment of the variations of LAI under heavy metal stress with the dominant points. (**a**) The locations of the dominant points in the original LAI curve and ratio curve: green areas represent the same location of the points; blue and red areas showed the different locations in the two kinds of curves; (**b**) the locations of the all dominant points in growth rate of rice roots (GR) for Area A, Area C and the differences between two areas.

After using the Harris algorithm, four points were selected form the LAI curve and ratio curve. Next, the points were filtered to reach the best effect, i.e., not only to obtain the smaller number but also to gain accuracy. All of the dominant points were placed in one figure, as shown in Figure 9a, where the green areas are the same location points of the LAI curve and LAI ratio curve, and the blue and red areas are the different location points of the two curves. To further verify the importance of these points, we used the LAI growth rate to distinguish them. Figure 9b illustrates the growth rate of the LAI in study area A and study area C and the LAI growth rate of A minus C, which follows the same trend. The five points, which are all of the dominant points, are shown in these growth rate curves; as shown in the Figure 9b, the LAI growth rate changed slowly and gently around the first point; the growth rate changed sharply near the second and fourth points, i.e., around those two points, the rice rapidly grew and reached its peak at the third point; finally, the growth rate exhibited a violent decline because rice no longer grows when close to death. Thus, with the analysis of these points in the growth rate curve, four points are finally obtained. The positions of the points are shown in Table 5.

**Figure 10:**
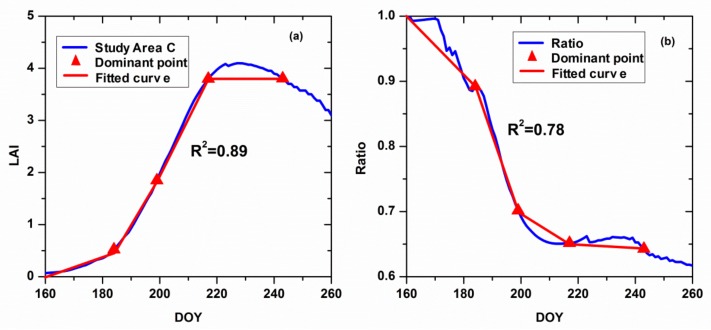
Assessment of the accuracy for the four dominant points. (**a**) shows the the fitted results of the LAI in study area C, (**b**) shows the ratio of the LAI in the two study areas.

In order to evaluate the accuracy of selecting the dominant points, we performed linear fitting on the dominant points and calculated the curve fitting correlation. The closer the fitted curve is to the original curve, the more accurate the selected dominant points are. Figure 10 shows the fitted results of the LAI and the ratio of the LAI in the two study areas. In terms of the LAI, after the dominant points were fitted, the R^2^ value is 0.89 between the fitted curve and original curve; for the ratio, the R^2^ value is 0.78 between the fitted curve and original ratio curve. Thus, it can be seen that the dominant points are accurate and reliable and can be used as the key period of the RS-WOFOST model.

### 4.3. Assessment of the Key Periods

The assessment of the key periods is conducted on the study area B. The time efficiencies and result accuracies of two period strategies were compared. In Period I, all the available CCD images (eight CCD images in total) in study area B during the entire rice growth period were used for assimilation. In Period II, the dominant points were chosen for assimilation. The simulated WRT of two period strategies were obtained and compared with the measured WRT. The accuracy and efficiency results of two period strategies are shown in Table 6. For simulation accuracy, Period I resulted in higher precision than Period II; overall, both periods resulted in good precision. However, with respect to time efficiency, Period II shortened the run time of the model operation by more than 50%, which is a clear advantage over Period I. Period II greatly reduced the time efficiency of the model while maintaining the accuracy of the model. It was proven that the selected key period was reasonable and practical; furthermore, the selected key period also provided the key period for the experimental field observations.

**Figure 11:**
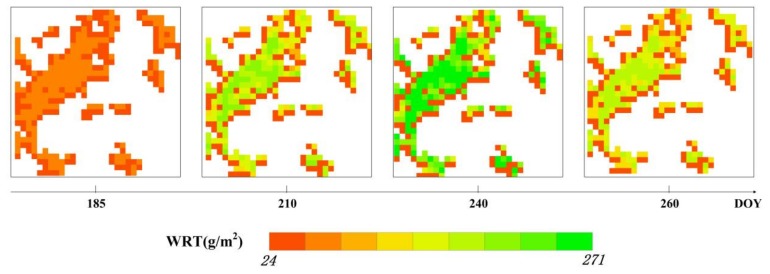
Spatial distribution of heavy metal stress level represented by continuous simulation of the WRT in study area B.

The LAI values calculated from the four CCD images based on the optimal time points were incorporated into the RS-WOFOST model, and thus, the spatio-temporal distribution of heavy metal stress in rice can be obtained by the RS-WOFOST model for each pixel for any day during the growing period. To clearly show the distribution of heavy metal stress in rice, four dates during each rice growth stage were chosen as being representative of the WRT distribution, which reflects the changes in the WRT during the entire growing season (Figure 11). As mentioned above, the irreversible effects of heavy metal suppress the normal growth of rice roots, which is clearly reflected in the negative correlation between heavy metal stress and the health of rice tissues. From the perspective of the growth mechanism, during the tillering stage and jointing–booting stage, rice tissues prioritize vegetative growth, and a large amount of water and nutrients are required, which is when many of the heavy metals enter into the root cells, which restrains and damages cell growth and affects the WRT; then, during the heading–flowering stage, rice tissues enter into the reproductive growth stage, which gives priority to grain growth; finally, during the ripening stage, the absorption of the roots declines, which reflects the ageing of the roots, and the effect of heavy metals on the rice decreases.

## 5. Discussion

The selection of the key period for the RS-WOFOST assimilation framework is very innovative. In this study, the optimal assimilation time points were determined using the Harris algorithm based on the LAI curves. It is very important for the RS-WOFOST assimilation framework applied at large scale, in monitoring the heavy metal stress in rice. This is because the more CCD images inserted, the longer the time that is required for the assimilation framework. However, the key period was determined based on the analysis of LAI, which was primarily applicable to the assimilation of LAI into WOFOST model. In application, considering the growing conditions and rice varieties, the key periods could also be suitable for rice which has similar growing conditions and the same rice varieties. The field measurement could also be conducted in the key period involved in this study. Considering the phenology of rice, the dates for the acquisition of the remote sensing images should be as close as possible to the four time points, but not confined to these time points. Meanwhile, further study is still needed to gain a better understanding of the role of each growth stage in stress monitoring when considering the phenology of rice [54,55]. The key period in this study was determined based on the rice growth cycle (approximately 100 days) in the study areas. If the conditions are different, such as the crop variety and region, the key period should be adjusted accordingly. In addition, the Harris detector method in this study is scale-dependent. In future work, an optimized multi-scale Harris method that can avoid the scale problems could be used for corner detection [56]. Also, a key period with equal intervals should be considered. However, this is only a suggestion as the result is unknown. On the contrary, the key period determined by the Harris algorithm based on the LAI curve resulted in a good outcome in that the key period improved the assimilation efficiency while maintaining its accuracy.

As a result, the verification in study area B indicated that assimilation with the key period could significantly improve the efficiency because it guarantees the accuracy, and shortens the run time of model operation by more than 50%, which leads to a more efficient and reliable choice of useful imagery to continuously monitor heavy metal stress in the study area with the RS-WOFOST model.

## 6. Conclusions

The objective of this study was to select the key periods for the RS-WOFOST assimilation framework. This process used the Harris algorithm based on the LAI curve under heavy metals stress. The result demonstrated that the key period of the RS-WOFOST model was consistent with the determination of the best observation stages, which means that selecting remotely sensed observations under heavy metals stress is more efficient and targeted. Considering the time-cost of regional-scale monitoring and the budget for field measurements, the selection of optimal time points is highly significant when monitoring heavy metals in rice on a large scale. It can also provide the optimal time for the field measurements in monitoring stress, in other words, on the premise of cost-savings, the measured data and remote sensing images can be effectively combined.

## Figures and Tables

**Table 1:** Content of heavy metals in the soil and rice in the study areas.

Heavy Metals	Background Value (bi) (mg/kg) ^1^	A (113°12′ E, 27°47′ N)		B (113°10′ E, 2740′ N)		C (113°2′ E, 27°50′ N)	
		Soil ^2^	Rice ^2^	Soil ^2^	Rice ^2^	Soil ^2^	Rice ^2^
**Cd**	1.43	0.84	0.82	2.26	7.23	3.28	5.90
**Pb**	82.78	78.33	10.60	91.05	15.18	120.75	36.73
**Hg**	0.2	0.18	0.04	0.3	0.04	0.51	0.06
**As**	19.11	10.23	5.39	18.33	6.29	18.15	7.04
**Pollution Index ^3^ (Cd)**		0.58		1.58		2.29	
**Pollution Level**		Safe		Level I		Level II	

Note: ^1^ The background values of the heavy metals were obtained from the Hunan Institute of Geophysical and Geochemical Exploration, China. ^2^ The heavy metals content in the sample plots were analyzed by the analysis and test Center, IEDA CAAS. ^3^ Pollution Index was calculated by the express Mean (Soil/Background values).

**Table 2:** Measured dry weight of rice roots (WRT) in the sample plots in the two study areas on four acquisition dates (DOY), unit of WRT is $g/m^{2}$

Sample Plot	184	211	241	260
A	34.4	245.7	262.5	201.3
C	31.0	223.6	239.3	189.4

**Table 3:** Stress factor (*f*) within each study area.

Study Area	Minimum	Maximum	Average
A	0.928	0.991	0.984
C	0.815	0.896	0.850

**Table 4:** Accuracy assessment of the WRT-WOFOST model.

Study Area	R^2^	MAE
A	0.988	0.214
C	0.97	0.199

**Table 5:** Position of the dominant points in two different curves and the determination of the dominant points.

Sample Plot	Serial Number of the Dominant Point			
	1	2	3	4
**Study Area C**	184 (DOY)	199	217	243
**Ratio (LAIC/LAIA)**	172	184	217	243
**Result**	184	199	217	243

**Table 6:** Comparison of the accuracy and efficiency between the two assimilation key periods.

Period	Time Points	Efficiency/s	R^2^	MAE
I	8	521	0.984	0.113
II	4	283	0.956	0.265
